# Feeding the world: impacts of elevated [CO_2_] on nutrient content of greenhouse grown fruit crops and options for future yield gains

**DOI:** 10.1093/hr/uhad026

**Published:** 2023-02-21

**Authors:** Nicholas H Doddrell, Tracy Lawson, Christine A Raines, Carol Wagstaff, Andrew J Simkin

**Affiliations:** NIAB, New Road, East Malling, Kent, ME19 6BJ UK; Department of Food and Nutritional Sciences, University of Reading, Whiteknights, Reading, Berkshire RG6 6DZ, UK; School of Life Sciences, University of Essex, Colchester CO4 4SQ, UK; School of Life Sciences, University of Essex, Colchester CO4 4SQ, UK; Department of Food and Nutritional Sciences, University of Reading, Whiteknights, Reading, Berkshire RG6 6DZ, UK; NIAB, New Road, East Malling, Kent, ME19 6BJ UK; School of Biosciences, University of Kent, Canterbury, United Kingdom CT2 7NJ, UK

## Abstract

Several long-term studies have provided strong support demonstrating that growing crops under elevated [CO_2_] can increase photosynthesis and result in an increase in yield, flavour and nutritional content (including but not limited to Vitamins C, E and pro-vitamin A). In the case of tomato, increases in yield by as much as 80% are observed when plants are cultivated at 1000 ppm [CO_2_], which is consistent with current commercial greenhouse production methods in the tomato fruit industry. These results provide a clear demonstration of the potential for elevating [CO_2_] for improving yield and quality in greenhouse crops. The major focus of this review is to bring together 50 years of observations evaluating the impact of elevated [CO_2_] on fruit yield and fruit nutritional quality. In the final section, we consider the need to engineer improvements to photosynthesis and nitrogen assimilation to allow plants to take greater advantage of elevated CO_2_ growth conditions.

## Introduction

**Figure 1:**
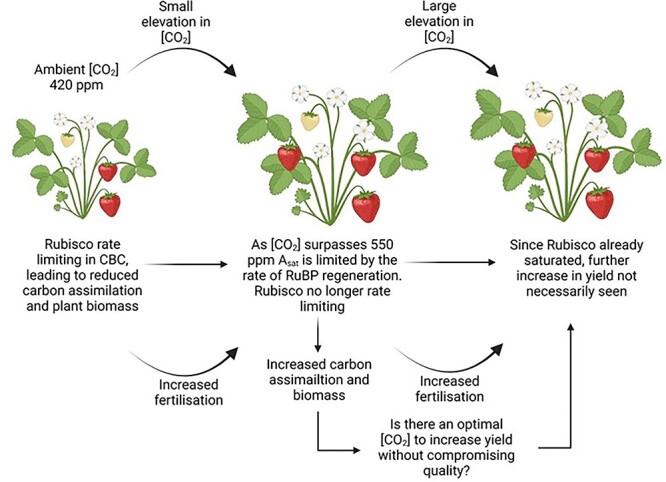
Schematic representation of elevated [CO_2_] on carbon assimilation. Created with BioRender.com

Elevated [CO_2_] (*e*[CO_2_]) has been shown to significantly improved light saturated photosynthetic carbon assimilation rates (*A_sat_*) by increasing the efficiency of Rubisco CO_2_ assimilation (carboxylation) over the alternate RuBP oxygenation (O_2_ assimilation), which results in enhanced growth and yield [1, 2] (Figure 1).

**Figure 2:**
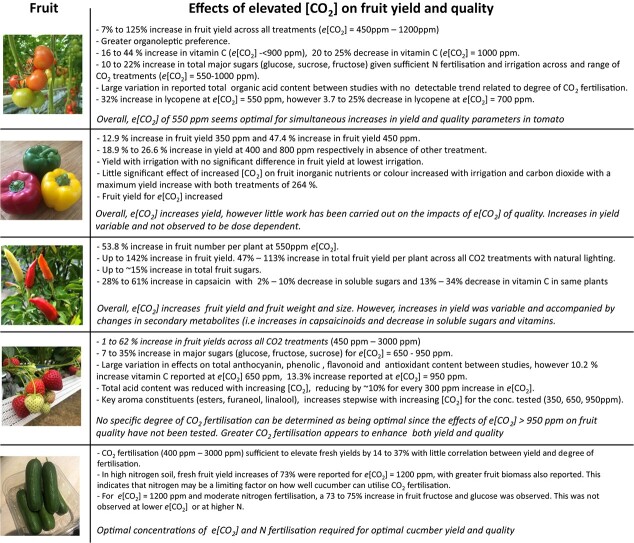
Effects of elevated [CO_2_] on yield and quality of fruiting crops. Created with BioRender.com

The majority of research evaluating the impact of *e*[CO_2_] on fruit crop production has been carried out in controlled environment conditions (chambers), polytunnels and commercial greenhouses where crops are grown in *e*[CO_2_], and focus almost exclusively on soft fruit such as strawberry, tomato and cucumber. Early work in the 1980’s suggested that *e*[CO_2_] increased the average yield of all plants tested by approximately 30%, with optional [CO_2_] concentration for growth and yield in the range of 700 to 900 ppm with concentration in excess of 1000 ppm having a negative impact on plant growth and yield [3–6]. In the case of vegetable cops, much of the work has been carried out in controlled environments, in which elevated [CO_2_] (800–900 ppm) increased lettuce, carrot, and parsley yield by 18%, 19%, and 17%, respectively in greenhouse grown crops. However, the yields of leek, chinese cabbage and celery were not significantly affected by increases in growth [CO_2_] concentration [7]. A meta-analysis of 107 selected articles showed that *e*[CO_2_] results in an increase in vegetable number (yield) by on average 32% and vegetable mass by 11% [8]. Furthermore, a meta-analysis of 57 articles consisting of 1015 observations found that *e*[CO_2_] has both positive and negative impacts on vegetable quality. For example, whilst concentrations of fructose (+14.2%), glucose (+13.2%), total soluble sugar (+17.5%), total antioxidant capacity (+59.0%), total phenols (+8.9%), total flavonoids (45.5%), vitamin C (+9.5%), and calcium (+8.2%) increased in the edible part of vegetables, protein (−9.5%,) nitrate (−18.0%), magnesium (−9.2%), iron (−16.0%), and zinc (−9.4%) decreased [9]. Moreover, a meta-analysis of legumes found a reduction in zinc and iron (and in non-legumes a reduction in protein) when plants were grown under *e*[CO_2_] (see Myers et al [10]). In 2018, Zhu et al [11] confirmed these results, and moreover demonstrated that rice grown under *e*[CO_2_] showed consistent declines in the quantities of vitamins B1, B2, B5, and B9 and, an increase in vitamin E. Finally, studies have shown that grains (wheat, rice, and barley), legumes, and maize-have a 4–10% reduction in iron concentrations of when grown under *e*[CO_2_] (~550 ppm) [12]. These results shown that *e*[CO_2_] can positively and negatively impact on legumes, grain and vegetables on a crop-by-crop basis and simultaneously alter quality attributes in the same harvestable material.

The aim of this review is to provide an overview of the current available data of the impact of elevated [CO_2_] on fruiting crops production in commercial growing systems. This paper examines these studies and the long-term implications of *e*[CO_2_] on the yield and quality of fruit required to feed a growing population. In the last section, we discuss the potential for designing crops for these new growing environments and allowing them to take full advantage of the introduced CO_2_, potentially increasing crop yield, reducing costs for commercial producers, and improving quality of the final product providing high nutritional value to consumers.

## Impact of elevated [CO_2_] on yield and quality of GREENHOSUE grown crops

### Impact of elevated [CO_2_] on solanaceous crops

Commercially, tomato crops are grown in greenhouses with *e*[CO_2_], in some cases as high as 2000 ppm. The effects of *e*[CO_2_] of fruit yield and quality has been extensively studied (Figure 2). Under *e*[CO_2_], tomato fruit yield increases ranged from 7% – 125% with [CO_2_] ranged from 450 ppm – 1200 ppm compared with plants grown under *a*[CO_2_]. An increase in the quantity of non-reducing sugars (glucose and fructose) has been reported [13–17] and fully ripe tomatoes grown in an *e*[CO_2_] were found to be preferable for consumption in sensory panels [13]. As liking sweetness has been shown to be a universal trait [18], it is possible that this increase in sugar is responsible for preference of the carbon enriched tomato fruits. An increase in vitamin C was also found between most studies [13, 15, 16, 19], potentially improving the health benefit gains from consumption of carbon-enriched grown tomatoes (Table 1). Vitamin C is an important dietary requirement and at high concentrations it has been used as a treatment for cancer, arteriosclerosis, and cardiovascular diseases [20–22]. These results suggest that increasing environmental [CO_2_] could contribute to an increase in Vitamin C improving their nutritional value for the consumer. However, growth at *e*[CO_2_] does not have the same impact on all species, as another studies in barley reported a significant decrease in Vitamin C content [23] highlighting the species–species response differences to *e*[CO_2_] and suggesting that high carbon growth environments may not always provide the best outcome for the consumer even though increases in yield maybe the producers primary concern (see Fenech et al. [24] and references therein).

Similarly, tomato fruit concentration of lycopene and β-carotene (pro-vitamin A) were found to increase in response to *e*[CO_2_] by as much as 30% and 70% respectively [13]. Rangaswamy et al. [25] reported an increase in carotenoid (+20%) and lycopene (+31%) in the fruits of tomato plants grown at 550 ppm [CO_2_], however carotenoid content decreased (− 12%) when the concentration was increased to 700 ppm*,* suggesting that the level of CO_2_ enrichment impacts fruit quality and careful consideration is needed to ensure an appropriate balance between levels of *e*[CO_2_] and final yield. Lycopene is an important phytonutrient, is sold commercially as a dietary supplement, and has been reported to possess anti-cancer properties and can improve cardiovascular health [26, 27].

β-carotene is the precursor for Vitamin A, also known as retinol. Vitamin A is an essential micronutrient playing important roles in growth and development, vision [28] and the immune system [29]. More than a third of all pre-school children and a significant number of pregnant women around the world are affected by Vitamin A deficiency, increasing the risk of night blindness and miscarriage [30, 31]. Importantly, most people suffering from a deficiency in Vitamin A show no clinical symptoms resulting in a phenomenon termed “Hidden Hunger” [32]. Production of crops with increased Vitamin A is therefore an important target for improving the diet and health of these at-risk groups; enhanced uptake of carbon may be a useful approach to achieve this. Increases in the Vitamin A precursor β-carotene has been observed in tomato fruit grown under *e*[CO_2_] of 800-900 ppm, in addition to a 28% increase in vitamin C at ripe stage and an ~8% increase in total soluble solids (Table 1). Zhang et al. [13], suggested that under these growth conditions, improved vitamin A and C and increased carotenoid content may be attainable.

Carotenoids are also the precursors of several flavour and aroma compounds. β-carotene is cleaved by carotenoid cleavage dioxygenases CCD1 and CCD4 [26, 33–35], to form the aromatic apocarotenoid β-ionone, which is important to tomato fruit flavour. Furthermore, lycopene, shown to increase under *e*[CO_2_] is cleaved by CCD1 to form several important flavour and aroma compounds including 6,10-dimethyl-3,5,9-undecatrien-2-one (pseudoionone [34];, 6-methyl-5-hepten-2-one (MHO [36]; and geranial [37]. MHO has been shown to be an important contributor to tomato fruit flavour [38, 39] and has also been shown to accumulate in tomato fruit with higher lycopene levels [40]. It is therefore apparent that growth in *e*[CO_2_] can increase a range of key flavour and nutraceutical precursor compounds present in tomato fruit; this phenomenon deserves further study, the optimal levels of [CO_2_] are currently not clear and more work is needed to better understand the relationship between CO_2_ assimilation carotenoid content, flavour and overall quality (Table 2).

Similar results have also been found in pepper crops, with yield increase of 12.9% – 370.2% reported when grown at *e*[CO_2_] of between 450 ppm – 1000 ppm (Table 2) with most other studies reporting yield increases in the range of 12.9% – 47.4% in the absence of other parameters [41–47]. However, it should be noted that growth at ~800 ppm *e*[CO_2_] was found to reduce sweet pepper total amino acid content by up to 29%, including reductions in the sweet tasting amino acids alanine and glycine, which could be detrimental to the perceived fruit flavour [42]. Yield was also found to vary with different irrigation programmes [41, 48], nitrogen sources [48], substrate salinity [42, 44] and pruning regimens [46]. Given that previous work in tomato has shown an increase in potential phytonutrients in fruit grown at 550 ppm and a decrease in those grown at 700 ppm, further research is needed to better identify the specific quantity of CO_2_ fertilisation necessary for maximally improved yield in solanaceous crops, especially when considering that CO_2_ uplift is often accompanied by additional treatments, such as increased nutrient and nitrogen fertilisation (Figure 2).

**Table 1:** Impact of elevated atmospheric [CO_2_] on yield and nutritional quality of tomato

**CO**_**2**_ **Treatment**	**Additional Treatment(s)**	**Fruit Yield**	**Fruit Quality**	**Ref**
510 ppm	N/A	9.9% increase in fruit yield.	N/A	[45]
590 ppm	root drying	Fruit dry weight not significantly affected by [CO_2_] across all irrigation treatments.	N/A	[155]
375 ppm – 675 ppm	Ozone treatment 80 nmol mol^−1^	24% increase in fruit yield. 31% decrease in fruit yield when exposed to ozone. Ozone and CO_2_ treated fruit yields were not significantly different to plants grown in ambient conditions.	N/A	[156]
550 ppm 700 ppm	N/A	54% increase in fruit yield at 550 ppm and 125% increase in fruit yield at 700 ppm.	1.4% – 11.4% decrease in total soluble solids, 27.3% – 31.8% decrease in total acids and 16.1% – 29.0% increase in vitamin C.	[19]
	+2 °C increase in temperature	18.4% – 21.4% increase in fruit yield due to increased [CO_2_].	10% increase in total sugars, 44% increase in vitamin C, 32% increase in lycopene at *e*[CO_2_] in absence of other treatments. *e*[CO_2_] rescues reduction in quality from increased temperature.	[25]
650 ppm 1000 ppm	N/A	17% increase in fruit yield at 650 ppm and 48% increase in fruit yield at 1000 ppm.	N/A	[3]
700 ppm	Doubled N fertilisation	N/A	13% – 25% decrease in fruit lycopene content across harvests with *e*[CO_2_]. 9% increase in fruit lycopene content with increased N fertilisation.	[157]
	UV-B exposure up to 1.744 kJ m^−2^	38% increase in fruit yield in absence of additional UV-B treatment, up to 46% increase in fruit yield with UV-B treatment.	Up to ~22% increase in soluble sugars, ~24% increase in organic acids, ~40 increase in vitamin C and ~ 47% increase in lycopene content of fruits grown under *e*[CO_2_] and UV-B treatment.	[15]
700 ppm 900 ppm	N/A	~30% increase in individual fruit weight.	~18% increase in vitamin C. ~Up to 20% reduction in major acids (citric, malic, oxalic). ~45% increase in sugars (glucose, fructose).	[16]
700 ppm 1000 ppm	N/A	32% increase in marketable fruit yield.	N/A	[158]
800 ppm	0–0.5 g N kg^−1^ soil. Soil water content 25% – 35%	Across all treatments, −3.3% – 28% increase in total fruit yield.	−17.9% – 11.9% increase in total fruit sugars and − 18.9% – 12.7% increase in total fruit acids across all treatments.	[159]
	Salinity treatments at 5–7 dS m^−1^	13% increase in yield in carbon-enriched atmosphere and 31% reduction in marketable fruit yield in increased salinity.	7% increase in total soluble solids. No significant change in citric acid content. Organoleptic qualities of tomatoes grown under increased salinity and CO_2_ found preferable in sensory trials.	[14]
	100 or 200 mg N kg^−1^ soil, 70% irrigation of control and root drying	8% increase in fresh fruit yield with increased [CO_2_].	No significant difference in total sugars, organic acid or fruit firmness for fruits grown in *e*[CO_2_].	[160]

(Continued)

**Table 1:** Continued

**CO**_**2**_ **Treatment**	**Additional Treatment(s)**	**Fruit Yield**	**Fruit Quality**	**Ref**
800 ppm – 900 ppm	N/A	N/A	~28% increase in vitamin C at ripe stage, ~8% increase in total soluble solids and no difference in total acids. Marked preference in sensory trials for fruits grown under enriched [CO_2_].	[13]
900 ppm	N/A	30% increase in marketable fruit yield.	N/A	[161]
	100 μmol s^−1^ m^−2^ supp lighting	12% – 15% increase in yield under supp lighting, 7% increase in yield in absence of additional treatment.	N/A	[162]
	N/A	22% increase in total fruit yield for plants grown in *e*[CO_2_].	N/A	[163]
1000 ppm	N/A	30% increase in total fresh fruit yield per plant.	N/A	[164]
		43% increase in total fruit yield.	No significant effect on fruit quality parameters.	[165]
		74.3% – 83.6% increase in tomato fresh weight per plant.	16.1% – 20.9% increase in total sugars. 20.0% – 24.7% decrease in vitamin C. 4.79% – 6.8% decrease in total acids.	[17]
		15.6% increase in fruit yield across 8 different cultivars.	N/A	[166]
1200 ppm	Salinity up to 4.58 x control	> 40% loss in dry fruit yield at highest salinity treatment completely offset by increased [CO_2_].	Increased salinity and [CO_2_] combined increases total sugar and acid content by up to ~30%.	[167]

In chili pepper, yield increases of 43.8% – 142% were reported for *e*[CO_2_] (in the range of 500 ppm – 1140 ppm). These yield increases were in part attributed to an increase in the size of fruits [49]. However, in controlled environments a 4°C increase in temperature decreased yield, even at *e*[CO_2_] (750 ppm) [50, 51], indicating that carbon enrichment is not sufficient to rescue yield where glasshouse facilities or growth tunnels experience periods of elevated temperature in an extreme climate change scenario. Carbon-enriched growth was found to increase the capsaicinoid content of fruits, resulting in an increase in Scoville Heat Units (SHU) [49, 52]. This approach therefore has potential for producing hotter varieties of chili, a growing and competitive market. However, at the same time Vitamin C concentration decrease by up to 15.84% [53], reducing potential health benefits gained from growing chilli plants under *e*[CO_2_]. (Table 2).

These reports suggest that the effects of growing crops in *e*[CO_2_] can have both a positive influence on yield and nutritional quality, however, growth at [CO_2_] levels above what is optimum can negatively impact some quality traits.

### Impact of elevated [CO_2_] on rosaceous crops

Rosaceous crop research in this area has focused primarily on cultivated strawberry with a small number of studies on raspberry and Nashi pear (Table 3). This is likely due to the relatively smaller size and rapid growth of strawberry compared to other commercially important rosaceous fruit species, such as tree fruits, like apple and cherry, and woody stemmed shrub fruits, like raspberry and blackberry. This makes strawberry a convenient plant to study as a rosaceous model. Furthermore, greater production of strawberry fruits would not only increase profits for growers but also decrease costs for consumers, increasing the availability of healthier options. Better access to such products through economic growth is strongly correlated to reduced micronutrient malnutrition or “hidden hunger” [54].

In cultivated strawberry, fresh fruit yield increases ranged from 1.0% – 62.0% in plants grown under atmospheric *e*[CO_2_] of 450 ppm – 3000 ppm, while dry fruit yield increased by up to 120% (Figure 2; Table 3). This has been directly linked to a 73% increase in assimilation rate of CO_2_ in strawberry leaves at optimal *e*[CO_2_] of 600 ppm [55–60]. Further investigation at a genetic level (through RNA seq analysis) revealed that 150 genes were upregulated in strawberry plants grown in an enriched-carbon atmosphere, with 14 of these being photosynthetic genes [60], suggesting that plants respond to these atmospheric increases by increasing their ability to assimilate the excess carbon.

Additional annual yield increases could be achieved by a two-week reduction in time to fruiting for plants grown in an enriched-carbon atmosphere [58, 61] increasing the field season and the period of productive (fruit) growth. Several fruit quality traits are also improved by growth at *e*[CO_2_]; increases in reducing sugars, and therefore sweetness index, were reported [62, 63] alongside reductions in organic acids [62]. These increases in sugar-acid ratio is highly favourable for a more pleasant perception of strawberry flavour by the consumer [18] and an increase in key volatile organic compounds, including furaneol, linalool and major esters, was also reported, further enhancing the “strawberry” aroma [62]. Growth in a carbon-enriched atmosphere therefore strongly enhances strawberry flavour and increases vitamin C (an important nutritional compound) by up to 13.3% alongside other antioxidant compounds [64, 65]. Growth in carbon-enriched atmospheres therefore simultaneously improves yield, flavour and health benefits of strawberry fruits, creating enormous potential for strategies involving enhanced photosynthesis of strawberry plants, including genetic manipulation. The greatest reported increase in fresh fruit yield where obtained when [CO_2_] was kept between 600 ppm – 1000 ppm [58], linking greater carbon assimilation to increased fresh fruit yield in strawberry and demonstrating an optimal degree of CO_2_ fertilisation for strawberries (Table 3).

**Table 2:** Impact of elevated atmospheric [CO_2_] on yield and nutritional quality of other Solanaceous crops

**Crop**	**CO**_**2**_ **Treatment**	**Additional Treatment(s)**	**Fruit Yield**	**Fruit Quality**	**Ref**
Sweet Pepper	350 ppm 450 ppm	N/A	12.9% increase in fruit yield 350 ppm and 47.4% increase in fruit yield 450 ppm.	N/A	[45]
	400 ppm – 800 ppm	20 mmol L^−1^ NaCl, foliar calcium treatment	18.9% to 26.6% increase in yield at 400 and 800 ppm respectively. Foliar calcium treatment had no impact on yield. *e*[CO_2_] rescued total yield loss from high salinity.	Little significant effect of increased [CO_**2**_] on fruit inorganic nutrients or colour.	[42]
	700 ppm	High/low irrigation and N treatments	Fruit yield for *e*[CO_2_] increased with irrigation with no significant difference in fruit yield at lowest irrigation.	N/A	[48]
	700 ppm – 750 ppm	N/A	18% – 22% increase in total fruit yield.	N/A	[46]
	800 ppm	Nitrogen source and saline treatment (8 and 25 mM NaCl)	8% and 22% increase in marketable fruit yield under salinity stress and unstressed respectively. 23% and 29% maximum increase in daily fruit harvest yield for 2 different nitrogen sources at low salinity.	N/A	[43,44,168]
	900 ppm	N/A	7% increase in early yielding fruits, no change in total fruit yield.	N/A	[162]
	367 ppm – 1000 ppm	Range of irrigation regimens	Yield increased with irrigation and carbon dioxide with a maximum yield increase with both treatments of 264%.	N/A	[41]
	1000 ppm	N/A	51% – 370% increase in fruit weight per plant.	N/A	[169]
	10 000 ppm	N/A	20% increase in fruit yield.	N/A	[61]
Chili pepper	380 ppm – 750 ppm	+2°C and + 4°C temperature elevation	Up to 41.9% increase in fruit diameter under both increased carbon dioxide and increased temperature.	27% – 44% increase in capsaicin across all treatments for 2 cultivars across 2 growth years.	[52]
	380 ppm – 750 ppm	+2°C and + 4°C temperature elevation	53.8% increase in fruit number at [CO_2_] = 550 ppm and ambient +2°C temperature, 12.3% decrease in fruit number per plant for [CO_2_] = 750 ppm and ambient +4°C temperature. Up to ~140% increase in fruit yield per plant for [CO_2_] = 550 ppm and ambient +2°C temperature, up to ~36% reduction in fruit yield per plant for [CO_2_] = 750 ppm and ambient +4°C temperature.	N/A	[50,51]
	380 ppm – 1140 ppm	N/A	Up to 88.5% increase in number of fruits per plant, up to 13.0% increase in fruit length, up to 15.0% increase in fruit width and up to 14.3% increase in pericarp thickness. Up to 142% increase in fruit yield.	No change in colour of ripe fruits. Up to 28.6% increase in capsaicinoids in ripe fruit.	[49,170]
	400 ppm – 900 ppm	Natural light (233 μmol m^−2^ s^−1^) and supplementary light (463 μmol m^−2^ s^−1^)	92% – 113% increase in total fruit yield per plant across all CO_2_ treatments with supplementary lighting relative to ambient control at 400 ppm. 47% – 113% increase in total fruit yield per plant across all CO_2_ treatments with natural lighting relative to ambient control at 400 ppm.	2% – 10% decrease in soluble sugars. 13% – 34% decrease in vitamin C in *e*[CO_2_]. 61% increase in capsaicin at [CO_2_] = 550 ppm, 49% – 61% decrease in capsaicin for [CO_2_] > 550 ppm.	[171]

**Table 2:** Continued

**Crop**	**CO**_**2**_ **Treatment**	**Additional Treatment(s)**	**Fruit Yield**	**Fruit Quality**	**Ref**
	1000 ppm	N/A	43.80% – 59.55% increase in fruit fresh weight per plant across 5 cultivars.	Up to ~15% increase in total fruit sugars. 11.84% – 15.84% decrease in fruit vitamin C, non-significant decrease in fruit titratable acids. Variable effects on inorganic nutrient concentrations. Fruit amino acids and fatty acids mostly reduced.	[53]
Eggplant	200 ppm – 3000 ppm	N/A	209% increase in fruit fresh weight and 134% increase in fruit dry weight.	N/A	[172]
	1000 ppm	N/A	31% increase in fruit yield across a full year of harvests.	N/A	[169]
	663 ppm	N/A	23.6% increase in fruit yield.	N/A	[45]

**Table 3:** Impact of elevated atmospheric [CO_2_] on yield and nutritional quality of Rosaceous crops

**Crop**	**CO**_**2**_ **Treatment**	**Additional Treatment(s)**	**Fruit Yield**	**Fruit Quality**	**Ref**
Strawberry	553 ppm	Nitrate treatment (4 x 10^–2 – 0^ mM)	42% increase in fresh fruit yield in *e*[CO_2_] at high N, 17% increase in fresh fruit yield in *e*[CO_2_] at low N.	N/A	[173]
	400 ppm, 650 ppm and 900 ppm	Ambient temperature (25°C) and elevated (30°C)	9.9% – 33.4% increase in total fruit yield at ambient temperature for cultivar “Albion”, 0.9% – 31.2% decrease in total fruit yield at ambient temperature for cultivar “San Andreas”. Elevated [CO_2_] rescues yield loss from elevated temperature.	Total fruit polyphenolic content, flavonoid content, monomeric anthocyanin content and antioxidant content increased in correlation with *e*[CO_2_] at both temperatures for multiple cultivars (~9% – ~325% increase overall increase at [CO_2_] = 900 ppm).	[56] [65]
	720 ppm	5°C increase in temperature, nitrate treatment (50 mL 0.1% NH_4_NO_3_ twice per week)	~120% increase in total fruit dry weight in *e*[CO_2_], ~73% increase in total fruit dry weight in *e*[CO_2_] with nitrogen treatment. No significant change in fruit yield for all other treatments individually and in combination.	48%, 21%, 36% and 18% decrease in fruit anthocyanin content, total phenolic content, total flavonoid content and total antioxidant content respectively at *e*[CO_2_]. 29% and 35% increase in fruit fructose and glucose respectively. 43% increase in total sugars.	[63]
	600 ppm – 1000 ppm	N/A	62% increase in total fruit yield in *e*[CO_2_].	N/A	[58]
	700 ppm – 1000 ppm	N/A	17.6% and 38.5% increase in individual fruit weight at [CO_2_] = ambient +300 ppm and [CO_2_] = ambient +600 ppm respectively.	7.0% – 25.9% increase in glucose, fructose and sucrose. 5.2% – 47.4% decrease in citric, malic and quinic acids. Stepwise increase in concentration of most key volatile esters and up to 115.0% and 149.6% increase in fruit furaneol and linalool content.	[62]
	700 ppm – 1000 ppm		N/A	13.3% increase in fruit ascorbic acid. Stepwise increase in antioxidant and flavonoid compounds with increasing carbon dioxide.	[64]
	700 ppm – 1000 ppm		5.4% and 12.7% increase in marketable fruit yield for cultivars “Irvine” and “Chandler” respectively.	N/A	[158]
	1000 ppm	N/A	47% increase in fruit number per plant, no significant change in individual fruit weight.	N/A	[55]
	900 ppm, 1500 ppm, 3000 ppm	N/A	31%, 43% and 51% increase in fruit yield at 900 ppm, 1500 ppm and 3000 ppm respectively.	N/A	[61]
Raspberry	436 ppm	N/A	12% increase in total berry yield and 5% increase in average individual berry weight.	N/A	[174]
Nashi Pear	700 ppm	Ambient +4°C temperature	16.6% increase in fruit weight with *e*[CO_2_]. Elevated [CO_2_] rescues yield loss from increased temperature.	Up to 15.9% reduction in fruit firmness with *e*[CO_2_]. Up to 22.5% increase in total soluble solids with no significant change in acidity with *e*[CO_2_].	[75]

### Impact of elevated [CO_2_] on cucurbitaceous crops

Cucumber is the most studied fruit crop of the cucurbitaceae in relation to growth in carbon-enriched atmospheres (Figure 2; Table 4). Improved carbon assimilation rates of up to 99% and 112% have been reported for cucumber and melon respectively when grown in *e*[CO_2_] [66, 67], demonstrating that growth in *e*[CO_2_] improves photosynthesis of cucurbitaceous crops.

**Table 4:** Impact of elevated atmospheric [CO_2_] on yield and nutritional quality of Cucurbitaceous crops

**Crop**	**CO**_**2**_ **Treatment**	**Additional Treatment(s)**	**Fruit Yield**	**Fruit Quality**	**Ref**
Cucumber	400 ppm, 625 ppm, 1200 ppm	2 mmol L^−1^, 7 mmol L^−1^, 14 mmol L^−1^ NO_3_^−^	Up to 73% increase in fresh fruit yield for plants grown at highest [CO_2_] versus plants grown at lowest [CO_2_] at greatest N fertilisation. No significant difference in yield for lower N fertilisation.	75% increase in fruit fructose, 73% increase in glucose at 7 mmol L^−1^ at highest [CO_2_]. No significant change in fruit titratable acidity. *e*[CO_2_] reduced dietary fibre by 13% – 18% across all fertilisation treatments. Up to 84% reduction in fruit nitrogenous compounds in *e*[CO_2_] across all nitrogen treatments.	[66] [68]
	400 ppm, 800 ppm, 1200 ppm	0.06 g N kg^−1^ soil (low N), 0.24 g N kg^−1^ soil (high N)	31% – 37% increase in fresh fruit yield for [CO_2_] = 800 ppm and 1200 ppm at low N. 71% – 106% increase in fresh fruit yield for [CO_2_] = 800 ppm and 1200 ppm at high N	Across both nitrogen treatments at [CO_2_] = 1200 ppm, fruit fructose was increased by 5% – 6%, fruit glucose was increased by 10% – 12% and starch was increased by 29% – 40%.	[70]
	364 ppm, 620 ppm	N/A	Up to 10.2% increase in individual fruit weight for August production in *e*[CO_2_]	No significant change in fruit dry matter content	[45]
	400–500 ppm	N/A	19% increase in fresh fruit yield at *e*[CO_2_]	N/A	[175]
	600–700 ppm	N/A	20% increase in fresh fruit yield at *e*[CO_2_]	N/A	[176]
	700 ppm	N/A	14.2% – 18.4% increase in fresh fruit yield at *e*[CO_2_] across two crop cycles.	Overall reduction in fruit inorganic nutrients (N, P, K, Ca, Mg).	[71]
	780 ppm	N/A	35% increase in fresh fruit yield in greenhouse supplemented with [CO_2_] versus control greenhouse.	N/A	[177]
	700 ppm – 1000 ppm	N/A	20% – 30% increase in marketable fruit yield across two growing seasons.	N/A	[158]
	900–1000 ppm	0.6°C – 1.8°C cooling	35.4% increase in dry fruit mass in cooled and *e*[CO_2_] conditions	N/A	[178]
	1000 ppm	N/A	8.9% increase in fruit weight but no significant change in fruit number at *e*[CO_2_]	N/A	[179]
	900 ppm, 1500 ppm, 3000 ppm	N/A	18.4% – 26.3% increase in fresh fruit yield across all CO_2_ elevations.	N/A	[61]
Melon	400 ppm, 800 ppm, 1200 ppm	0, 25, 50 mmol NaCl	Up to 29% increase in fruit yield in all *e*[CO_2_] at no additional salinity. Elevated [CO_2_] partially rescues yield loss from salinity (by up to 18%) but is insufficient to fully mitigate yield loss.	N/A	[67]
	1000 ppm	N/A	13% increase in muskmelon fruit number and 8% increase in muskmelon fruit weight during summer production under *e*[CO_2_]	N/A	[169]
Squash	700–1000 ppm	N/A	15.5% – 19.7% increase in total marketable yield across 2 growing seasons.	N/A	[158]

In cucumber (*Cucumis sativus*), fruit yield increases for plants grown in enriched-carbon atmospheres ([CO_2_] = 450 ppm – 3000 ppm) ranged between 16.2% and 41% in the absence of other parameters that could alter fruit yield. In high nitrogen supplemented fertilisation, fruit yield was as high as 106% when grown under *e*[CO_2_] of 800 ppm [68], indicating the potential of increased nitrogen fertilisation alongside [CO_2_] enrichment to unlock the greatest yield increases in cucumber. Interestingly, when grown under *e*[CO_2_] of 1200 ppm with the addition of high nitrogen fertilisation treatment, studies found a yield increase between 71% – 73% [66, 68], which was lower than the 106% for plants grown at *e*[CO_2_] of 800 ppm. Concentrations of [CO_2_] above optimal reduced stomatal density, stomatal conductance (*gs*), the maximum carboxylation rate (*Vc*max) and the maximum photosynthetic electron transport rate (*J*max) [69]. This suggests that an optimal concentration of atmospheric [CO2] exists for maximum yield returns and deserves further investigation. There is large variation between studies on how cucumber fruit quality is impacted by carbon-enriched growth. Fructose and glucose were reported to increase by 6% and 12% in one study [68] and by 75% and 73% respectively in another [70]. The inorganic nutrient content of fruits was also reported to decrease in fruits grown in *e*[CO_2_], however only phosphorus showed a significant reduction in multiple cycles [71]. These data do suggest that *e*[CO_2_] may enhance fruit flavour and fruit yield at the expense of nutritional value.

### Impact of elevated [CO_2_] on yield and quality of fruiting trees

Sweet clonal cherry (*Prunus avium* L.) plants were grown for 19 months in climate-controlled greenhouses at ambient (1994–358 ppm; 1995–360 ppm) or *e*[CO_2_] (700 ppm). Elevated [CO_2_] treatment increased photosynthesis and dry matter production, leaf (55%) and stem (61%), after two months at 700 ppm, however, this initial stimulation is not sustained. Photosynthetic rates were less after 10 months of growth than after 2 months of growth, and only small increases in dry mass are still evident after 10-months, suggesting that sweet cherry acclimates to *e*[CO_2_] due to long-term exposure [72]. Due to the young nature of plants studied compared with fully grown mature trees (deciduous tree 15–32 m in height and with a trunk up to 1.5 m in circumference [73, 74], no information is available to determine the impacts of *e*[CO_2_] on fruit yield or quality. In Nashi pear, a CO_2_-enriched atmosphere of 700 ppm increased fruit weight, diameter and length along with a 22.5% increase in Brix, (a key measure of sweetness for marketable fruit [75]). However, this also resulted in a reduction in fruit firmness demonstrating that improvements in yield can be nullified by negative impacts on fruit quality (Table 3).

While these studies are limited in, they do indicate the potential of CO_2_-enriched growth for improving photosynthesis, increasing yield and quality of tree crops. However, they also suggest that some crops, especially perennial crops, may become acclimated to higher [CO_2_] and any gains may be lost over time.

### Does increasing carbon assimilation increase environmental tolerances?

The work presented above also suggest that increasing CO_2_ uptake could have other benefits. It is notable that growth of fruit crops in carbon enriched atmospheres has a similar effect of protecting against environmental stresses, such as drought and elevated temperature, that may become increasingly common due to climate change as plants genetically engineered to increase carbon assimilation. For example, in melon (*Cucumis melo*), growing plants in *e*[CO_2_] has been shown to mitigate yield losses from increased salinity [67], and in sweet pepper, *e*[CO_2_] of 800 ppm was sufficient to rescue any significant yield loss of total and marketable fruits from salinity stress (20 mmol L^−1^ NaCl) [42]. It could be hypothesised that increasing CO_2_ assimilation increases sugar and chlorophyll content triggering salt tolerance. However, it should be noted that these results are not universally translatable. Gray et al. [76] demonstrated in soybean that *e*[CO_2_] was insufficient to protect yields from drought conditions triggered by higher temperatures demonstrating that benefits in some crops may not be translatable across all crops of agronomical importance. Furthermore, in tomato plant Zhou et al. [77] showed that plants grown in *e*[CO_2_] were more sensitive to combined drought and heat stress; *e*[CO_2_] drives *gs* and transpiration reducing net photosynthesis and therefore productivity, which is concerning given that greenhouses tend to have elevated temperatures compared to the external environment due to the nature of their construction, glass and metal, and therefore *e*[CO_2_] in an enclosed system may negatively impact on yields if water supplies are limiting. This demonstrates that irrigation within greenhouse environments is an essential element and adjusting water regimes to maintain productivity and optimise water-use efficiency.

It is also important to note that it is the increase in atmospheric [CO_2_] that causes the increase in air temperature (along with associated stresses) by absorbing energy and preventing it from being radiated out into space (see [78, 79]); as such one might view that the cause cannot mitigate its own effects, however, in some crops where both [CO_2_] and temperature increase simultaneously, yields were maintained compared with data where temperature is increased in the absence of *e*[CO_2_] leading to yield loss and these results cannot be ignored, but a better understanding of the impact of cause and effect climate change on crop yields needs to be researched, otherwise, the logic consequences would be further increase amounts of [CO_2_] in the atmosphere to increase crop tolerance against the effects of ever-increasing temperatures.

Interestingly, some parallels do exist between photosynthetically genetically modified crops and increased tolerance to salinity. In Arabidopsis, over-expression of Sedoheptulose-1,7-bisphosphatase (SBPase), which enhances CO_2_ assimilation rates by increasing the regeneration of the Rubisco substrate RuBP [80], enhances salt tolerance through increases in sucrose, starch and chlorophyll content were reported [81]. This suggests that increasing photosynthetic rates, either through increasing the availability of [CO_2_] for photosynthesis or increasing the plants’ ability to assimilate [CO_2_] under ambient conditions could have a similar protective effect. It would be interesting to explore if increased carbon assimilation rates, through atmospheric manipulation or genetic modification, can have a positive impact on crop resistance to high salt environments and other abiotic stresses in large field trials or commercial greenhouses. There is currently evidence that over-expressing the Calvin-Benson cycle (CBC) enzyme SBPase can increase tolerance to chilling stress in tomato [82] and the expression of the cyanobacterial CBC bifunctional fructose-1,6-bisphosphatases/Sedoheptulose-1,7-bisphosphatase enzyme in soybean prevent yield loss under high temperature [83]. Köhler et al. [83] concluded that the manipulation of CO_2_ uptake could mitigate against the effects of global increases in temperature under *e*[CO_2_]. This may be deemed especially important given the expected impact of global climate change. This suggests that increasing carbon assimilation through manipulation of photosynthesis [84, 85] can have similar outputs to improved photosynthesis through growth in an enriched carbon atmosphere and further demonstrates the viability of this approach for improvement of yield and quality in fruiting crops. This must be studied considering the recent work showing that improved carbon assimilation also results in improved nutrient uptake and an increase in NUE [86].

### Future opportunities

As [CO_2_] surpasses 550 ppm, *A_sat_* will be limited by the rate of RuBP regeneration rather than Rubisco activity suggesting there is scope to improve plant photosynthesis to increase yield in greenhouse environments where CO_2_ is routinely increased to 1000 ppm or more for short periods of time. These short time-periods are furthermore unpredictable and chaotic given that greenhouses must be vented, due to external environmental conditions, to maintain, as close as possible, optimal growing conditions i.e temperature and humidity inside the growth facility. Furthermore, the [CO_2_] dosing capacity must be economically beneficial, especially given the chaotic nature of CO_2_ loss to the environment during periods of venting. As dosing increases, costs go up accordingly determined by the cost of CO_2_. Moreover, at some point, there is a price limit where the supplemental cost of CO_2_ increases to a point where costs cannot be recovered by the selling price of the product. In the last year, CO_2_ costs have increased for £100 per tonne to as much as £3000 per tonne [87, 88]. Therefore, future options that maximize the ability of the crop to take full benefit of the *e*[CO_2_], or maintaining higher yields when CO_2_ costs are unmanageable become more important.

**Figure 3:**
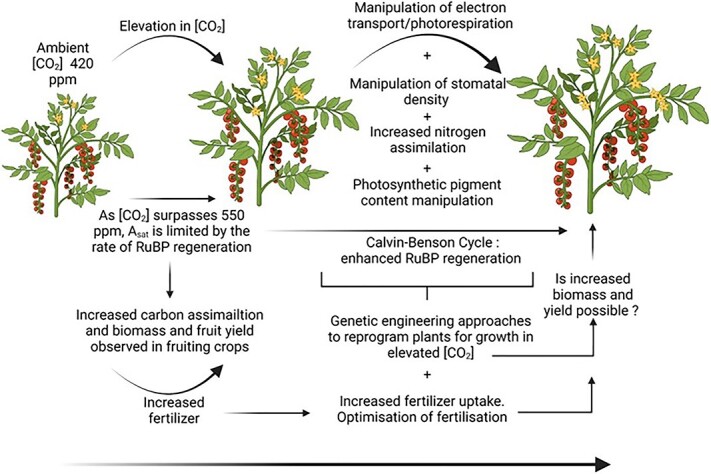
Effects of elevated [CO_2_] on yield of fruiting crops and a representation of the potential for the manipulation of plant material for further yield increases. Created with BioRender.com

Araus et al [89], noted that canopy photosynthesis holds a crucial place in a context of yield gains through photosynthetic improvement, which requires additional factors including the availability and uptake of nutrients, such as nitrogen, irrigation, the transport of photoassimilates and sink-source balance. As such, in addition to improving photosynthetic rates via CO_2_ supplementation, the improvement of other plant processes such as N uptake, non-foliar photosynthesis, stomatal function, and rubisco(activase) thermotolerance so that crops are better adapted for growth in [CO_2_] enriched environments such as greenhouses are discussed below (Figure 3). These works will also need to account for changes to the landscape of greenhouse crop cultivation, such as a move to vertical farming, changes in growth medium from soil to substrates such as coir (derived from coconut husks) or rockwool [90]. It is estimated that more than 50% of strawberry production occurs in substrate rather than soil [91]. Coir is often used as it has been shown to retains water more efficiently than soil, so strawberry plants require less frequent watering improving water use efficiency. Coir also has a high level of aeration, which is ideal for strawberries’ whose root systems require a lot of oxygen. More recent developments in hydroponics [92] and aeroponics [93], will impact on irrigation, fertiliser regimes and N uptake.

#### Nitrogen use efficiency (NUE)

With regards to fruit quality, this is a complex trait that may not be simply attributed to enhanced carbon assimilation. More research is needed to link increased assimilate, with assimilate distribution and transport, NUE to better understand the sink-source relationship in any given crop, which can vary significantly across varieties and crop types. NUE is determined by yield per unit of available N in the growth medium (i.e often coir in greenhouse grown crops). Plants with higher NUE may allocate N toward both the photosynthetic complexes (i.e N is major component of chlorophyll; total N allocated to Rubisco 18.2 ± 6.2% [94];) and/or toward the development of additional sinks. The second definition of NUE could be described as the efficiency with which N is applied to soils, (through artificial means in greenhouse crops), is taken up by plants and converted to usable products (i.e. biomass, grain yield). This can be manipulated through breeding to identify new varieties with high NUE uptake from selected growing mediums or through engineering nitrogen symbiosis (Figure 3). Recently, scientists reported the engineering nitrogen-fixation into non-legume cereal crops by enabling them to interact with soil bacteria to convert N from the air into ammonia fertiliser [95]. These works could firstly reduce the reliance on commercial synthetic fertilisers and secondly provide alternate sources of N that along with improvements to carbon assimilation, foliar or non-foliar, co-contribute to improving photosynthesis and yields in crops (Figure 3).

A recent review has identified a number of targets in the literature to improve N uptake, assimilation and remobilisation through genetic manipulation (see [96] for review). One of these, the over-expression of the nitrate transporter (NRT2.3) was shown to increase nitrate concentrations in tomato increasing biomass and fruit weight [97]. More recently, the transcription factor DREB1C has been identified as a regulator of NUE by controlling the expression of several important growth-related genes including the rubisco small subunit 3 (RBCS3), nitrate transporters (NRT1.1B, NRT2.4), nitrate reductase (NR2) and the flowering regulator (FTL3). Once over-expressed (OE), OsDREB1C increased the abundance of photosynthetic pigments, plants were shown to have about one-third more chloroplasts, 38% more rubisco and improved photosynthesis and N uptake. The OE of OsDREB1C resulted in a > 40% increase in grain yield in elite rice varieties and an ~20% increase in wheat yields, while in Arabidopsis, a significant increase in biomass [98]. Many of these identified genes have potential for improving NUE in fruiting crops grown in *e*[CO_2_]. A recent report of a large grain rice cultivar, Akita 63, having a high yield due to an enlarged sink capacity without and photosynthesis improvement. However, this work demonstrated that source capacity was strongly limiting the yield potential under high N fertilization. These authors suggested that enhancing photosynthesis is an important step to further increase yield of current high-yielding cultivars [99]. This work can be extrapolated that engineering NUE and photosynthesis in plants grown at *e*[CO_2_] could provide a step-change in yields in greenhouse cultivated crops.

#### Genetic variation in photosynthetic traits in crops and wild relatives

Methods of improving these traits including breeding, by exploiting the potential of crop wild relatives as a source of new traits, and/or the genetic manipulation/genome editing of specific traits. There is already evidence that substantial genetic variation exists within wild relatives of fruiting crops [100–102], which are now studied as a source of crop improvement in various breeding programs [103]. Further evidence that even in elite material, significant variation is observed in photosynthetic traits. For example, *V*_*c*max_, *J_max_* and *A_sat_*, indicators of photosynthetic potential, have been shown to vary by as much as 30% in the flag leaves of recent breeding lines of spring and winter wheats [104–106]. Similarly, several quantitative trait loci for photosynthetic efficiency have been identified in elite rice material, including the identification of important transcription factors [107, 108]. This work in wheat and rice is promising, demonstrating the potential for breeding new varieties better adapted to changing growth conditions, however it is unclear if such strategies will work in horticultural crops. In the case of tomato, there is considerable variation within the wild and elite varieties to suggest that such breeding strategies could be used to enhanced yield and quality [109, 110]. See Sharwood et al [111] for review (Figure 3).

In transgenic rice, overproducing Rubisco, increases the biomass production and yield under high N fertilization in paddy fields suggesting that the development of new rice varieties with both high photosynthesis and large sink capacity is essential [99]. Furthermore, genes encoding thermostable variants of Rubisco activase (thermos-Rca) have been identified in wild rice relatives*.* When over-expressed in domesticated rice, thermos-Rca was sufficient to enhance carbohydrate accumulation and improve yields after periodic exposure to elevated temperatures (+45°C) throughout the vegetative phase [112, 113]. Thermostable Rca have been identified in Thermophilic cyanobacteria, bacteria that thrive in high-temperature environments, making them a potential source of novel genes for engineering crops for growth at higher temperatures [114]. Improving the thermal tolerance of rubisco activase, either through breeding with wild populations or genetic engineering, could aid greenhouse grown crops better tolerate the elevated temperatures that often occur during the growing season (Figure 3).

#### Genetic engineering of photosynthetic traits in crops

Increasing the expression of enzymes and/or proteins involved in the regeneration of RuBP, CO_2_ transport or chloroplast electron transport have previously been shown to enhance photosynthetic efficiency and increases in yield [84, 85, 115–117]. However, once again, it cannot be ignored that much of this work has focused on non-fruiting crops, such as Arabidopsis, tobacco, wheat and rice, (see Simkin et al. [84] for review), grown in controlled conditions, performed in pots, in soil or in the field with controlled irrigation, which is not typical of global agriculture. Furthermore, work carried out in tomato, over-expression of sedoheptulose-1,7-bisphosphatase, involved in RuBP regeneration, did not report on fruit yield [82]. These data indicating that more work is required to understand how these manipulations would impact fruiting crops grown in tightly controlled environments.

One potential target for genetic manipulation is the starch synthesis enzyme adenosine diphosphate glucose pyrophosphorylase (AGPase); increasing AGPase activity has potential to increase starch accumulation for growth. Increased accumulation of starch has been shown to have little negative feedback on photosynthesis [118] and increased AGPase activity in the chloroplast would increase the strength of the transient starch pool, which acts as a sink in the chloroplast. Reduced sink capacity does induce negative feedback on photosynthesis and can limit photosynthesis even in favourable conditions (e.g. elevated [CO_2_]) [119], suggesting that increasing the sink may allow for greater CO_2_ assimilation in supplemented [CO_2_] growth environments.

Although genetic manipulation has the potential to further increase yields in crops grown in enriched [CO_2_] environments, allowing them to take better advantage of supplemental CO_2,_ increasing net photosynthetic rates and associated yields (Figure 3), it should also be noted that some reports have suggested that increases in yield in genetically enhanced photosynthetic crops are likely not uniquely down to increases in carbon assimilation but a combination of factors; for example improvements in carbon uptake allow for an increase in N assimilation [120]. Furthermore, it has also been reported that such increase in yield from enhanced photosynthetic efficiency critically rely on the availability and uptake of water and nutrients (for review see [121, 122]), therefore, genetic engineering as an approach alone may be limiting if other aspects of crop cultivation, such as irrigation, planting regimes, fertilisation (i.e NUE) and growth media (i.e soil, coir, rockwool), are not taken into account and co-optimised.

#### Non-foliar photosynthesis

Leaves are not the only location within the plant where photosynthesis occurs, with evidence of photosynthesis in petioles and stems [123, 124], and fruit [124] that may provide significant and alternative sources of photo-assimilates essential for optimal yield. Assimilation of atmospheric CO_2_ is dependent on the number and behaviour of stomata, and the stems of many plants have stomata distributed along the epidermis [125, 126] and an evaluation of the photosynthetic activity in stems of various plants accounted for up to 4% of the total photosynthetic activity [127]. Furthermore, Hu et al. demonstrated the importance of stem photosynthesis to yield in cotton; maintaining the stem in darkness reduced seed weight by 16% [128] showing the stem provides photoassimilates for plant development and growth.

As previously noted, many fruiting crops produce green fruit containing all the necessary proteins and enzymes to carry out photosynthesis [127, 129, 130] that may provide significant and alternative sources of photoassimilates essential for optimal yield and quality [124]. Tomato fruit photosynthesis contributes to net sugar accumulation and growth and previous work concluded that tomato fruit photosynthesis contributes between 10% and 15% of the total fixed carbon of the fruit [127, 131] [132],. It should be noted that, unlike many crops, cucumber fruit remain green through to maturity, have stomata (suggesting they perform gas exchange to drive photosynthesis), and have a similar surface area to an expanded leaf [130]. It has previously been reported that cucumber fruit had high photosynthetic and respiratory rates [133] and contribute approximately 9.4% of their own carbon requirements [130]. It should be noted that in fruit with stomata, such as cucumber, there are two potential major sources of CO_2_. Firstly, Rubisco assimilates atmospheric [CO_2_] through the stomatal pores, leading to the production of sugars via the CBC and secondly, CO_2_ released by mitochondrial respiration is re-fixed (recycling photosynthesis) [125, 134]. Whilst this confirms that photosynthesis occurs in fruits, the extent and importance is not clear. In *e*[CO_2_], it seems plausible that cucumber fruit photosynthesis may contribute directly to fruit size (and therefore yield by weight) and quality through their ability to directly access carbon in an enriched atmosphere via their stomata (for a review fruit photosynthesis, see [124, 135]. Therefore, increasing carbon capture by non-foliar tissues has the potential to significantly impact yield and combined with an increase N uptake (i.e. slow release fertilizers [136]) to balance the increased carbon uptake, and optimised irrigation regimes has the potential to maximise such yield gains.

## Conclusions

These data show that the yield of fruiting crops benefit from growth in supplemented atmospheres, although, some data suggests that increase in yield can come at the expense of quality traits. It is therefore essential to determine the optimal [CO_2_] concentrations on a crop-by-crop basis, to maximise productivity. An evaluation of fruit quality under these conditions has also been shown to be highly variable between treatments and difference are observed between cultivars with the same treatment suggesting that much more research is required to identify the specific mechanisms behind changes in fruit quality. In the case of soft fruit production in greenhouse environments, it will be important to determine if the quality of fruit harvested early in the season differs from that of fruit harvested later in the season when plants have spent a more significant period of time exposed to *e*[CO_2_] growth conditions. Cherry for instance, when grown under prolonged periods of *e*[CO_2_], acclimates to prolonged exposure and initial significant gains in yield observed after two months are less detectable after ten months and are not significantly different to control plants grown at *a*[CO_2_] [72]. This may in one respect account for differences in nutritional quality observed in fruit grown in similar conditions in different studies (i.e. fruit harvested at different times in the study) where additional fertilizer treatments aren’t provided.

Increases in yield associated with *e*[CO_2_] controlled environments may be about more than additional carbon. Controlled environments also allow the regulation of transpiration (e.g. by controlling vapour pressure deficit) and therefore water uptake and the inclusion of additional fertilisation (specifically N). Breeding new varieties adapted to these growth conditions may also be more amenable given the hostility towards genetically modified crops. A recent review noted that new phenomics, genomics, and bioinformatics tools make it possible to harness the untapped potential of crop genetic resources (including wild relatives) to create combinations of traits to enhance yield in high [CO_2_] controlled environments [137].

Breeding alone may not be sufficient to adapt all varieties, or all crops, to high [CO_2_] growing environments traditionally used in greenhouses. However, over the last several decades, agricultural research has adopted technologies such as genetic engineering and “genome editing” to improve traits in key crops that could be useful in these circumstances [85, 138–140]. These include advances in the tools available to carry out this work, including vectors for multiple gene insertion [141–145] and tissue specific promoters [146–150]. If the promise of these biotechnology programs is to be realized, it will be necessary to address the public perception of genetic modification and genome editing technologies to gain greater acceptance.

Genetic manipulation, may need to go beyond the direct manipulation of carbon assimilation in leaves [84, 180], but focus on the manipulating and control of stomatal function [151, 152], the manipulation of pigments complexes in ripening fruit [153], enhancement of light capture by the leaves through the manipulation of chlorophyll distribution and form [154] and importantly look a methodologies for increasing N uptake via transgenic [96] or traditional means (improved fertilization regimes).

It should also be noted that the introduction of new growing, hydroponics, aquaponics and aeroponics may require further study, to breed and adapt or engineer plants root architecture for these new growth media. In conclusion, greenhouse cultivation offers the opportunity to manipulate growing atmosphere, lights and VPD for improved yields and we can now look at the opportunities to breed and engineer plants specifically optimised for these conditions.

## Acknowledgments

This research is funded by the Biotechnology and Biological Sciences Research Council (BBSRC) Collaborative Training Partnerships (CTP) for Fruit Crop Research in partnership with NIAB EMR and Reading University. N.H.D was supported by “Realising increased photosynthetic efficiency to increase strawberry yields” (BBSRC, BB/S507192/1) awarded to A.J.S. A.J.S is supported by the Growing Kent and Medway Program, UK; Ref 107139. 

## Author Contributions

N.H.D and A.J.S drafted and wrote the manuscript with input from T.L, C.A.R and C.W who also edited the final version.

## Conflict of interests

The authors declare no competing interests.

## References

[1] Ainsworth EA , Rogers A. The response of photosynthesis and stomatal conductance to rising [CO_2_]: mechanisms and environmental interactions. Plant Cell Environ. 2007;30:258–70. 17263773 10.1111/j.1365-3040.2007.01641.x

[2] Kimball BA . Crop responses to elevated CO_2_ and interactions with H_2_O, N, and temperature. Curr Opin Plant Biol. 2016;31:36–43. 27043481 10.1016/j.pbi.2016.03.006

[3] Kimball BA , Mitchell ST. CO_2_ enrichment of tomatoes in unventilated greenhouses in an arid climate. Acta Hortic. 1978;87:131–8.

[4] Mortensen LM . Review: CO2 enrichment in greenhouses. Crop responses. Sci Hortic. 1987;33:1–25.

[5] Idso SB . Three phases of plant response to atmospheric CO_2_ enrichment. Plant Physiol. 1988;87:5–7. 16666125 10.1104/pp.87.1.5 PMC1054688

[6] Prior SA , Rogers HH, Runion GB et al. Effects of free-air CO_2_ enrichment on cotton root growth. Agric For Meteorol. 1994;70:69–86.

[7] Mortensen LM . Effects of elevated CO_2_ concentrations on growth and yield of eight vegetable species in a cool climate. Sci Hortic. 1994;58:177–85.

[8] Dong J , Gruda N, Li X et al. Sustainable vegetable production under changing climate: the impact of elevated CO_2_ on yield of vegetables and the interactions with environments-a review. J Clean Prod. 2020;253:119920.

[9] Dong J , Gruda N, Lam SK et al. Effects of elevated CO_2_ on nutritional quality of vegetables: a review. Front Plant Sci. 2018;9:924. 30158939 10.3389/fpls.2018.00924 PMC6104417

[10] Myers SS , Zanobetti A, Kloog I et al. Increasing CO_2_ threatens human nutrition. Nature. 2014;510:139–42. 24805231 10.1038/nature13179 PMC4810679

[11] Zhu C , Kobayashi K, Loladze I et al. Carbon dioxide (CO_2_) levels this century will alter the protein, micronutrients, and vitamin content of rice grains with potential health consequences for the poorest rice-dependent countries. Sci Adv. 2018;4:1–8. 10.1126/sciadv.aaq1012 PMC5966189 29806023

[12] Smith MR , Golden CD, Myers SS. Potential rise in iron deficiency due to future anthropogenic carbon dioxide emissions. Geohealth. 2017;1:248–57. 32158990 10.1002/2016GH000018 PMC7007116

[13] Zhang Z , Liu L, Zhang M et al. Effect of carbon dioxide enrichment on health-promoting compounds and organoleptic properties of tomato fruits grown in greenhouse. Food Chem. 2014;153:157–63. 24491715 10.1016/j.foodchem.2013.12.052

[14] Sánchez-González MJ , Sánchez-Guerrero MC, Medrano E et al. Carbon dioxide enrichment: a technique to mitigate the negative effects of salinity on the productivity of high value tomatoes. Span J Agric Res. 2016;14:e0903.

[15] Li F , Wang J, Chen Y et al. Combined effects of enhanced ultraviolet-B radiation and doubled CO_2_ concentration on growth, fruit quality and yield of tomato in winter plastic greenhouse. Frontiers of Biology in China. 2007;2:414–8.

[16] Shahidul Islam M , Matsui T, Yoshida Y. Effect of carbon dioxide enrichment on physico-chemical and enzymatic changes in tomato fruits at various stages of maturity. Sci Hortic. 1996;65:137–49.

[17] Khan I , Azam A, Mahmood A. The impact of enhanced atmospheric carbon dioxide on yield, proximate composition, elemental concentration, fatty acid and vitamin C contents of tomato (*Lycopersicon esculentum*). Environ Monit Assess. 2013;185:205–14. 22382378 10.1007/s10661-012-2544-x

[18] Drewnowski A , Mennella JA, Johnson SL et al. Sweetness and food preference. J Nutr. 2012;142:S1142–8. 10.3945/jn.111.149575 PMC3738223 22573785

[19] Mamatha H , Rao NK, Laxman RH et al. Impact of elevated CO2 on growth, physiology, yield, and quality of tomato (Lycopersicon esculentum mill) cv. Arka Ashish. Photosynthetica. 2014;52:519–28.

[20] Du J , Cullen JJ, Buettner GR. Ascorbic acid: chemistry, biology and the treatment of cancer. Biochimica et Biophysica Acta (BBA) - Reviews on Cancer. 2012;1826:443–57. 22728050 10.1016/j.bbcan.2012.06.003 PMC3608474

[21] Leong SY , Oey I. Effects of processing on anthocyanins, carotenoids and vitamin C in summer fruits and vegetables. Food Chem. 2012;133:1577–87.

[22] Da Silva Dias JC . Nutritional and health benefits of carrots and their seed extracts. Food Nutr Sci. 2014;05:2147–56.

[23] Robinson JM , Sicher RC. Antioxidant levels decrease in primary leaves of barley during growth at ambient and elevated carbon dioxide levels. Int J Plant Sci. 2004;165:965–72.

[24] Fenech M , Amaya I, Valpuesta V et al. Vitamin C content in fruits: biosynthesis and regulation. Frontiers in Plant Science (Review). 2019;9:1–21. 10.3389/fpls.2018.02006 PMC6353827 30733729

[25] Rangaswamy TC , Sridhara S, Ramesh N et al. Assessing the impact of higher levels of CO_2_ and temperature and their interactions on tomato (*Solanum lycopersicum* L.). Plan Theory. 2021;10:256. https://www.mdpi.com/2223-7747/10/2/256. 10.3390/plants10020256 PMC7911991 33525663

[26] Simkin AJ . Carotenoids and apocarotenoids in planta: their role in plant development, contribution to the flavour and aroma of fruits and flowers, and their nutraceutical benefits. Plan Theory. 2021;10:2321. 10.3390/plants10112321 PMC8624010 34834683

[27] Story EN , Kopec RE, Schwartz SJ et al. An update on the health effects of tomato lycopene. Annu Rev Food Sci Technol. 2010;1:189–210. 22129335 10.1146/annurev.food.102308.124120 PMC3850026

[28] Rando RR . The chemistry of vitamin a and vision. Angew Chem Int Ed. 1990;29:461–80.

[29] West CE , Rombout JH, van der Zijpp AJ et al. Vitamin a and immune function. Proc Nutr Soc. 1991;50:251–62. https://www.ncbi.nlm.nih.gov/pubmed/1749794. 1749794 10.1079/pns19910035

[30] WHO . In: WHO Global Database on Vitamin A Deficiency, ed. Global Prevalence of Vitamin a Deficiency in Populations at Risk 1995–2005. Geneva: World Health Organisation, 2005.

[31] WHO . World Health Organisation : Micronutrient Deficiencies. Geneva: World Health Organisation, 2019.

[32] Hodge J . Hidden hunger: Approaches to tackling micronutrient deficiencies in Nourishing millions: Stories of change in nutrition. In: Gillespie S, Hodge J, Yosef S, Pandya-Lorch R, eds. International Food Policy Research Institute (IFPRI): Washington, D.C., 2016,35–46 Ch. 4.

[33] Simkin AJ , Underwood BA, Auldridge M et al. Circadian regulation of the PhCCD1 carotenoid cleavage dioxygenase controls emission of beta-ionone, a fragrance volatile of petunia flowers. Plant Physiol. 2004;136:3504–14. 15516502 10.1104/pp.104.049718 PMC527150

[34] Simkin AJ , Schwartz SH, Auldridge M et al. The tomato carotenoid cleavage dioxygenase 1 genes contribute to the formation of the flavor volatiles b-ionone, pseudoionone, and geranylacetone. Plant J. 2004;40:882–92. 15584954 10.1111/j.1365-313X.2004.02263.x

[35] Zhang X , Pei J, Zhao L et al. Overexpression and characterization of CCD4 from Osmanthus fragrans and β-ionone biosynthesis from β-carotene in vitro. J Mol Catal B Enzym. 2016;134:105–14.

[36] Vogel JT , Tan B-C, McCarty DR et al. The carotenoid cleavage dioxygenase 1 enzyme has broad substrate specificity, cleaving multiple carotenoids at two different bond positions. J Biol Chem. 2008;283:11364–73. 18285342 10.1074/jbc.M710106200

[37] Ilg A , Beyer P, Al-Babili S. Characterization of the rice carotenoid cleavage dioxygenase 1 reveals a novel route for geranial biosynthesis. FEBS J. 2009;276:736–47. 19120446 10.1111/j.1742-4658.2008.06820.x

[38] Baldwin EA , Scott JW, Shewmaker CK et al. Flavor trivia and tomato aroma: biochemistry and possible mechanisms for control of important aroma components. Hort Science. 2000;35:1013–22.

[39] Buttery RG , Teranishi R, Ling LC et al. Quantitative and sensory studies on tomato paste volatiles. J Agric Food Chem. 1990;38:336–40.

[40] Simkin AJ , Gaffé J, Alcaraz JP et al. Fibrillin influence on plastid ultrastructure and pigment content in tomato fruit. Phytochemistry. 2007;68:1545–56. 17466343 10.1016/j.phytochem.2007.03.014

[41] Rezende FC , Frizzone JA, Oliveira RFD et al. CO_2_ and irrigation in relation to yield and water use of the bell pepper crop. Sci Agric. 2003;60:7–12.

[42] Piñero MC , Pérez-Jiménez M, López-Marín J et al. Fruit quality of sweet pepper as affected by foliar Ca applications to mitigate the supply of saline water under a climate change scenario. J Sci Food Agric. 2018;98:1071–8. 28722753 10.1002/jsfa.8557

[43] Porras ME , Lorenzo P, Medrano E et al. Photosynthetic acclimation to elevated CO_2_ concentration in a sweet pepper (*Capsicum annuum*) crop under Mediterranean greenhouse conditions: influence of the nitrogen source and salinity. Funct Plant Biol. 2017;44:573–86. 32480589 10.1071/FP16362

[44] Porras ME , Lorenzo P, Medrano E et al. Sweet pepper grown under salinity stress as affected by CO_2_ enrichment and nitrogen source. Acta Hortic. 2017;1170:805–12.

[45] Nederhoff EM . Effects of CO_2_ concentration on photosynthesis, transpiration and production of greenhouse fruit vegetable crops. Phd Thesis, Wageningen University and Research, 1994.

[46] Alonso FJ , Lorenzo P, Medrano E et al. Greenhouse sweet pepper productive response to carbon dioxide enrichment and crop pruning. Acta Hortic. 2012;927:345–51.

[47] Aloni B , Karni L. Effects of CO_2_ enrichment on yield, carbohydrate accumulation and changes in the activity of antioxidative enzymes in bell pepper (*Capsicum annuum* L.). J Hortic Sci Biotechnol. 2002;77:534–40.

[48] Penuelas J , Biel C, Estiarte M. Growth, biomass allocation and phenology responses of pepper to elevated CO_2_ concentrations and different water and nitrogen supply. Photosynthetica. 1995;31:91–9.

[49] Garruña-Hernández R , Monforte-González M, Canto-Aguilar A et al. Enrichment of carbon dioxide in the atmosphere increases the capsaicinoids content in habanero peppers (*Capsicum chinense* Jacq.). J Sci Food Agric. 2013;93:1385–8. 23124470 10.1002/jsfa.5904

[50] Das S , Das R, Kalita P et al. Growth responses of hot chilli (*Capsicum chinense* jacq.) to elevated carbon dioxide and temperature. Journal of Experimental Biology and Agricultural Sciences. 2020;8:434–40.

[51] Das S , Das R, Kalita P et al. Developmental processes in hot chilli (*Capsicum chinense* Jacq.) as affected by elevated carbondioxide and temperature. Plant Physiology Reports. 2020;25:386–94.

[52] Das S , Das R, Hemendra C et al. Interactive effect of elevated carbondioxide and high temperature on quality of hot chilli (*Capsicum chinense* Jacq.). Int J Trop Agric. 2016;34:1977–81.

[53] Azam A , Hameed A, Khan I. Impact of elevated atmospheric carbon dioxide on yield, vitamin c, proximate, fatty acid and amino acid composition of capsicum (*capsicum Annuum*). Environmental Pollution and Protection. 2017;2:153–67.

[54] Gödecke T , Stein AJ, Qaim M. The global burden of chronic and hidden hunger: trends and determinants. Global Food Security. 2018;17:21–9.

[55] Sung FJM , Chen JJ. Gas exchange rate and yield response of strawberry to carbon dioxide enrichment. Sci Hortic. 1991;48:241–51.

[56] Balasooriya HN , Dassanayake KB, Seneweera S et al. Interaction of elevated carbon dioxide and temperature on strawberry (*Fragaria × ananassa*) growth and fruit yield. International Journal of Biological, Life and Agricultural Sciences. 2018;11:1–9.

[57] Bunce JA . Seasonal patterns of photosynthetic response and acclimation to elevated carbon dioxide in field-grown strawberry. Photosynth Res. 2001;68:237–45. 16228346 10.1023/A:1012928928355

[58] Bushway LJ , Pritts MP. Enhancing early spring microclimate to increase carbon resources and productivity in june-bearing strawberry. J Am Soc Hortic Sci. 2002;127:415–22.

[59] Keutgen N , Chen K, Lenz F. Responses of strawberry leaf photosynthesis, chlorophyll fluorescence and macronutrient contents to elevated CO_2_. J Plant Physiol. 1997;150:395–400.

[60] Li X , Zhao J, Shang M et al. Physiological and molecular basis of promoting leaf growth in strawberry (*Fragaria ananassa* Duch.) by CO_2_ enrichment. Biotechnology & Biotechnological Equipment. 2020;34:905–17.

[61] Enoch HZ , Rylski I, Spigelman M. CO_2_ enrichment of strawberry and cucumber plants grown in unheated greenhouses in Israel. Sci Hortic. 1976;5:33–41.

[62] Wang S , Bunce J. Elevated carbon dioxide affects fruit flavor in field-grown strawberries (*Fragaria × ananassa* Duch). J Sci Food Agric. 2004;84:1464–8.

[63] Sun P , Mantri N, Lou H et al. Effects of elevated CO_2_ and temperature on yield and fruit quality of strawberry (*Fragaria × ananassa* Duch.) at two levels of nitrogen application. PLoS One. 2012;7:1–12. 10.1371/journal.pone.0041000 PMC3404062 22911728

[64] Wang SY , Bunce JA, Maas JL. Elevated carbon dioxide increases contents of antioxidant compounds in field-grown strawberries. J Agric Food Chem. 2003;51:4315–20. 12848504 10.1021/jf021172d

[65] Balasooriya HN , Dassanayake KB, Seneweera S et al. Impact of elevated carbon dioxide and temperature on strawberry polyphenols. J Sci Food Agric. 2019;99:4659–69. 30906993 10.1002/jsfa.9706

[66] Dong J , Li X, Chu W et al. High nitrate supply promotes nitrate assimilation and alleviates photosynthetic acclimation of cucumber plants under elevated CO_2_. Sci Hortic. 2017;218:275–83.

[67] Mavrogianopoulos GN , Spanakis J, Tsikalas P. Effect of carbon dioxide enrichment and salinity on photosynthesis and yield in melon. Sci Hortic. 1999;79:51–63.

[68] Dong J , Xu Q, Gruda N et al. Elevated and super-elevated CO_2_ differ in their interactive effects with nitrogen availability on fruit yield and quality of cucumber. J Sci Food Agric. 2018;98:4509–16. 29479715 10.1002/jsfa.8976

[69] Xu M . The optimal atmospheric CO2 concentration for the growth of winter wheat (Triticum aestivum). J Plant Physiol. 2015;184:89–97. 26253981 10.1016/j.jplph.2015.07.003

[70] Dong J-L , Li X, Nazim G et al. Interactive effects of elevated carbon dioxide and nitrogen availability on fruit quality of cucumber (Cucumis sativus L.). J Integr Agric. 2018;17:2438–46.

[71] Segura ML , Parra JF, Lorenzo P et al. The effects of CO_2_ enrichment on cucumber growth under greenhouse conditions. Acta Hortic. 2001;559:217–22.

[72] Atkinson CJ , Taylor JM, Wilkins D et al. Effects of elevated CO_2_ on chloroplast components, gas exchange and growth of oak and cherry. Tree Physiol. 1997;17:319–25. 14759855 10.1093/treephys/17.5.319

[73] Vignati E , Lipska M, Dunwell JM et al. Fruit development in sweet cherry. Plan Theory. 2022;11:1531. 10.3390/plants11121531 PMC9227597 35736682

[74] Vignati E , Lipska M, Dunwell JM et al. Options for the generation of seedless cherry, the ultimate snacking product. Planta. 2022;256:90. 36171415 10.1007/s00425-022-04005-y PMC9519733

[75] Han J-H , Cho JG, Son IC et al. Effects of elevated carbon dioxide and temperature on photosynthesis and fruit characteristics of ‘Niitaka’ pear *(Pyrus pyrifolia* Nakai). Hortic Environ Biotechnol. 2012;53:357–61.

[76] Gray SB , Dermody O, Klein SP et al. Intensifying drought eliminates the expected benefits of elevated carbon dioxide for soybean. Nature Plants. 2016;2:16132. 27595230 10.1038/nplants.2016.132

[77] Zhou R , Yu X, Wen J et al. Interactive effects of elevated CO_2_ concentration and combined heat and drought stress on tomato photosynthesis. BMC Plant Biol. 2020;20:260. 32505202 10.1186/s12870-020-02457-6 PMC7276063

[78] Stips A , Macias D, Coughlan C et al. On the causal structure between CO_2_ and global temperature. Sci Rep. 2016;6:21691. 26900086 10.1038/srep21691 PMC4761980

[79] Koutsoyiannis D , Kundzewicz ZW. Atmospheric temperature and CO_2_: hen-or-egg causality? Sci. 2020;2:83. https://www.mdpi.com/2413-4155/2/4/83.

[80] Simkin AJ , Lopez-Calcagno PE, Davey PA et al. Simultaneous stimulation of sedoheptulose 1,7-bisphosphatase, fructose 1,6-bisphophate aldolase and the photorespiratory glycine decarboxylase H-protein increases CO_2_ assimilation, vegetative biomass and seed yield in Arabidopsis. Plant Biotechnol J. 2017;15:805–16. 27936496 10.1111/pbi.12676 PMC5466442

[81] Chen Y , Jiang Y, Chen Y et al. Uncovering candidate genes responsive to salt stress in *Salix matsudana* (Koidz) by transcriptomic analysis. PLoS One. 2020;15:1–23. 10.1371/journal.pone.0236129 PMC7410171 32760076

[82] Ding F , Wang M, Zhang S et al. Changes in SBPase activity influence photosynthetic capacity, growth, and tolerance to chilling stress in transgenic tomato plants. Sci Rep. 2016;6:32741. 27586456 10.1038/srep32741 PMC5009361

[83] Kohler IH et al. Expression of cyanobacterial FBP/SBPase in soybean prevents yield depression under future climate conditions. J Exp Bot. 2017;68:erw435–726. 10.1093/jxb/erw435 PMC5441901 28204603

[84] Simkin AJ , Lopez-Calcagno PE, Raines CA. Feeding the world: improving photosynthetic efficiency for sustainable crop production. Journal of Experimantal Botany. 2019;70:1119–40. 10.1093/jxb/ery445 PMC6395887 30772919

[85] Simkin AJ . Genetic engineering for global food security: photosynthesis and biofortification. Plan Theory. 2019;8:586. https://www.mdpi.com/2223-7747/8/12/586. 10.3390/plants8120586 PMC6963231 31835394

[86] Sekhar KM , Kota VR, Reddy TP et al. Amelioration of plant responses to drought under elevated CO_2_ by rejuvenating photosynthesis and nitrogen use efficiency: implications for future climate-resilient crops. Photosynth Res. 2021;150:21–40. 32632534 10.1007/s11120-020-00772-5

[87] ECIU . Gas prices adding £1.7 billion to cost of beer and bangers. In: Smeeton G, ed. Informed Debate on Energy and Climate Change. Energy and Climate Intelligence Unit. London, UK: Energy and Climate Intelligence Unit (ECIU), 2022.

[88] ECIU . Farming, Fertilisers and Fossil Fuels. In: How the Gas Crisis Is Squeezing Britain's Farmers. Energy and Climate Intelligence Unit. London, UK: Energy and Climate Intelligence Unit (ECIU), 2022,1–22.

[89] Araus JL , Sanchez-Bragado R, Vicente R. Improving crop yield and resilience through optimization of photosynthesis: panacea or pipe dream? J Exp Bot. 2021;72:3936–55. 33640973 10.1093/jxb/erab097

[90] Xiong J , Tian Y, Wang J et al. Comparison of coconut coir, rockwool, and peat cultivations for tomato production: nutrient balance, plant growth and fruit quality. Front Plant Sci. 2017;8:1327. 28824665 10.3389/fpls.2017.01327 PMC5539188

[91] Robinson, Boyer L, Feng W, Gulbis N et al. The use of arbuscular mycorrhizal fungi to improve strawberry production in coir substrate. Front Plant Sci. 2016;7:1–9. 27594859 10.3389/fpls.2016.01237 PMC4991251

[92] Nguyen NT , McInturf SA, Mendoza-Cózatl DG. Hydroponics: a versatile system to study nutrient allocation and plant responses to nutrient availability and exposure to toxic elements. J Vis Exp. 2016;54317:55–66. 10.3791/54317 PMC5091364 27500800

[93] Eldridge BM , Manzoni LR, Graham CA et al. Getting to the roots of aeroponic indoor farming. New Phytol. 2020;228:1183–92. 32578876 10.1111/nph.16780

[94] Luo X , Keenan TF, Chen JM et al. Global variation in the fraction of leaf nitrogen allocated to photosynthesis. Nat Commun. 2021;12:4866. 34381045 10.1038/s41467-021-25163-9 PMC8358060

[95] Haskett TL , Paramasivan P, Mendes MD et al. Engineered plant control of associative nitrogen fixation. Proc Natl Acad Sci U S A. 2022;119:1–9. 10.1073/pnas.2117465119 PMC9169844 35412890

[96] Lebedev VG , Popova AA, Shestibratov KA. Genetic engineering and genome editing for improving nitrogen use efficiency in plants. Cell. 2021;10:3303. 10.3390/cells10123303 PMC8699818 34943810

[97] Fu Y , Yi H, Bao J et al. LeNRT2.3 functions in nitrate acquisition and long-distance transport in tomato. FEBS Lett. 2015;589:1072–9. 25819437 10.1016/j.febslet.2015.03.016

[98] Wei S , Li X, Lu Z et al. A transcriptional regulator that boosts grain yields and shortens the growth duration of rice. Science. 2022;377:eabi8455. 35862527 10.1126/science.abi8455

[99] Makino A , Suzuki Y, Ishiyama K. Enhancing photosynthesis and yield in rice with improved N use efficiency. Plant Sci. 2022;325:1–9. 10.1016/j.plantsci.2022.111475 36167261

[100] Aflitos S , Schijlen E, de Jong H et al. Exploring genetic variation in the tomato (*Solanum section Lycopersicon*) clade by whole-genome sequencing. The Plant journal : for cell and molecular biology. 2014;80:136–48. 25039268 10.1111/tpj.12616

[101] Sahu KK , Chattopadhyay D. Genome-wide sequence variations between wild and cultivated tomato species revisited by whole genome sequence mapping. BMC Genomics. 2017;18:430. 28576139 10.1186/s12864-017-3822-3 PMC5455116

[102] Blanca J , Montero-Pau J, Sauvage C et al. Genomic variation in tomato, from wild ancestors to contemporary breeding accessions. BMC Genomics. 2015;16:257. 25880392 10.1186/s12864-015-1444-1 PMC4404671

[103] Cockerton HM , Karlström A, Johnson AW et al. Genomic informed breeding strategies for strawberry yield and fruit quality traits. Front Plant Sci. 2021;12:1–16. 10.3389/fpls.2021.724847 PMC8525896 34675948

[104] Driever SM , Lawson T, Andralojc PJ et al. Natural variation in photosynthetic capacity, growth, and yield in 64 field-grown wheat genotypes. J Exp Bot. 2014;65:4959–73. http://www.ncbi.nlm.nih.gov/pubmed/24963002. 24963002 10.1093/jxb/eru253 PMC4144772

[105] Silva-Pérez V , de Faveri J, Molero G et al. Genetic variation for photosynthetic capacity and efficiency in spring wheat. J Exp Bot. 2020;71:2299–311. 31565736 10.1093/jxb/erz439 PMC7134913

[106] Sales CRG , Molero G, Evans JR et al. Phenotypic variation in photosynthetic traits in wheat grown under field versus glasshouse conditions. J Exp Bot. 2022;73:3221–37. 35271722 10.1093/jxb/erac096 PMC9126738

[107] Adachi S , Yamamoto T, Nakae T et al. Genetic architecture of leaf photosynthesis in rice revealed by different types of reciprocal mapping populations. J Exp Bot. 2019;70:5131–44. 31257428 10.1093/jxb/erz303 PMC6793464

[108] Adachi S , Yoshikawa K, Yamanouchi U et al. Fine mapping of carbon assimilation rate 8, a quantitative trait locus for flag leaf nitrogen content, stomatal conductance and photosynthesis in rice. Frontiers in Plant Science (Original Research). 2017;8:60. 10.3389/fpls.2017.00060 PMC5282472 28197156

[109] de Oliveira Silva FM , Lichtenstein G, Alseekh S et al. The genetic architecture of photosynthesis and plant growth-related traits in tomato. Plant Cell Environ. 2018;41:327–41. 29044606 10.1111/pce.13084

[110] Lana-Costa J , de Oliveira Silva FM, Batista-Silva W et al. High photosynthetic rates in a *Solanum pennellii* chromosome 2 qtl is explained by biochemical and photochemical changes. Front Plant Sci. 2020;11:1–15. 32595679 10.3389/fpls.2020.00794 PMC7303335

[111] Sharwood RE , Quick WP, Sargent D et al. Mining for allelic gold: finding genetic variation in photosynthetic traits in crops and wild relatives. J Exp Bot. 2022;73:3085–108. 35274686 10.1093/jxb/erac081

[112] Scafaro AP , Atwell BJ, Muylaert S et al. A thermotolerant variant of rubisco activase from a wild relative improves growth and seed yield in rice under heat stress. Front Plant Sci. 2018;9:1–11. 30524456 10.3389/fpls.2018.01663 PMC6256286

[113] Scafaro AP , Gallé A, van Rie J et al. Heat tolerance in a wild Oryza species is attributed to maintenance of rubisco activation by a thermally stable rubisco activase ortholog. New Phytol. 2016;211:899–911. 27145723 10.1111/nph.13963

[114] Ogbaga CC , Stepien P, Athar H-U-R et al. Engineering rubisco activase from thermophilic cyanobacteria into high-temperature sensitive plants. Crit Rev Biotechnol. 2018;38:559–72. 28937283 10.1080/07388551.2017.1378998

[115] López-Calcagno PE , Brown KL, Simkin AJ et al. Stimulating photosynthetic processes increases productivity and water-use efficiency in the field. Nature Plants. 2020;6:1054–63. 32782406 10.1038/s41477-020-0740-1

[116] Driever SM et al. Increased SBPase activity improves photosynthesis and grain yield in wheat grown in greenhouse conditions. Philos Trans R Soc B. 2017;372:1730. 10.1098/rstb.2016.0384 PMC5566882 28808101

[117] Raines CA , Cavanagh AP, Simkin AJ. In: Ruban A, Murchie E, Foyer C, eds. Chapter 9. Improving Carbon Fixation in Photosynthesis in Action 1. London, UK: Elsevier, Academic Press, 2022.

[118] Petreikov M , Yeselson L, Shen S et al. Carbohydrate balance and accumulation during development of near-isogenic tomato lines differing in the AGPase-L1 allele. J Am Soc Hortic Sci. 2009;134:134–40.

[119] Ainsworth EA , Bush DR. Carbohydrate export from the leaf: a highly regulated process and target to enhance photosynthesis and productivity. Plant Physiol. 2011;155:64–9. 20971857 10.1104/pp.110.167684 PMC3075787

[120] Yoon D-K , Ishiyama K, Suganami M et al. Transgenic rice overproducing rubisco exhibits increased yields with improved nitrogen-use efficiency in an experimental paddy field. Nature Food. 2020;1:134–9. 10.1038/s43016-020-0033-x 37127998

[121] Sinclair TR , Rufty TW, Lewis RS. Increasing photosynthesis: unlikely solution for world food problem. Trends Plant Sci. 2019;24:1032–9. 31488354 10.1016/j.tplants.2019.07.008

[122] Wu A , Hammer GL, Doherty A et al. Quantifying impacts of enhancing photosynthesis on crop yield. Nature Plants. 2019;5:380–8. 30962528 10.1038/s41477-019-0398-8

[123] Hibberd JM , Quick WP. Characteristics of C4 photosynthesis in stems and petioles of C3 flowering plants. Nature. 2002;415:451–4. 11807559 10.1038/415451a

[124] Simkin AJ , Faralli M, Ramamoorthy S et al. Photosynthesis in non-foliar tissues: implications for yield. Plant J. 2020;101:1001–15. 31802560 10.1111/tpj.14633 PMC7064926

[125] Aschan G , Pfanz H. Non-foliar photosynthesis – a strategy of additional carbon acquisition. Flora. 2003;198:81–97.

[126] Ávila E , Herrera A, Tezara W. Contribution of stem CO2 fixation to whole-plant carbon balance in nonsucculent species. Photosynt. 2014;52:3–15.

[127] Hetherington SE , Smillie RM, Davies WJ. Photosynthetic activities of vegetative and fruiting tissues of tomato. J Exp Bot. 1998;49:1173–81.

[128] Hu Y-Y , Zhang YL, Luo HH et al. Important photosynthetic contribution from the non-foliar green organs in cotton at the late growth stage. Planta. 2012;235:325–36. http://www.jstor.org/stable/43564330. 21904871 10.1007/s00425-011-1511-z

[129] Carrara S , Pardossi A, Soldatini GF et al. Photosynthetic activity of ripening tomato fruit. Photosynt. 2001;39:75–8.

[130] Sui X , Shan N, Hu L et al. The complex character of photosynthesis in cucumber fruit. J Exp Bot. 2017;68:1625–37. 28369547 10.1093/jxb/erx034 PMC5441898

[131] Obiadalla-Ali H , Fernie AR, Lytovchenko A et al. Inhibition of chloroplastic fructose 1,6-bisphosphatase in tomato fruits leads to decreased fruit size, but only small changes in carbohydrate metabolism. Planta. 2004;219:533–40. 15060828 10.1007/s00425-004-1257-y

[132] Tanaka A , Fujita K, Kikuchi K. Nutrio-physiological studies on the tomato plant. Soil Science and Plant Nutrition. 1974;20:57–68.

[133] Todd GW , Bean RC, Propst B. Photosynthesis & respiration in developing fruits II. Comparative rates at various stages of development. Plant Physiol. 1961;36:69–73. 16655473 10.1104/pp.36.1.69 PMC406092

[134] Millar AH , Whelan J, Soole KL et al. Organization and regulation of mitochondrial respiration in plants. Annu Rev Plant Biol. 2011;62:79–104. 21332361 10.1146/annurev-arplant-042110-103857

[135] Blanke MM . Fruit Photosynthesis. In: Ruban A, Garab G, (ed.), Photosynthesis: Mechanisms and Effects. Dordrecht, Netherlands: Springer, 1998.

[136] Li T , Zhang W, Yin J et al. Enhanced-efficiency fertilizers are not a panacea for resolving the nitrogen problem. Glob Chang Biol. 2018;24:e511–21. 28973790 10.1111/gcb.13918

[137] Reynolds M , Atkin OK, Bennett M et al. Addressing research bottlenecks to crop productivity. Trends Plant Sci. 2021;26:607–30. 33893046 10.1016/j.tplants.2021.03.011

[138] Wilson F , Harrison K, Armitage AD et al. CRISPR/Cas9-mediated mutagenesis of phytoene desaturase in diploid and octoploid strawberry. Plant Methods. 2019;15:45. 31068975 10.1186/s13007-019-0428-6 PMC6495592

[139] Aglawe SB , Barbadikar KM, Mangrauthia SK et al. New breeding technique "genome editing" for crop improvement: applications, potentials and challenges. Biotech. 2018;8:336. 10.1007/s13205-018-1355-3 PMC6056351 30073121

[140] Georges F , Ray H. Genome editing of crops: a renewed opportunity for food security. GM Crops and Food. 2017;8:1–12. 28075688 10.1080/21645698.2016.1270489 PMC5592977

[141] Exposito-Rodriguez M , Laissue P, López-Calcagno P et al. Development of pGEMINI, a plant gateway destination vector allowing the simultaneous integration of two cDNA via a single LR-clonase reaction. Plants (Basel). 2017;6:55. 29137147 10.3390/plants6040055 PMC5750631

[142] Engler C , Gruetzner R, Kandzia R et al. Golden gate shuffling: a one-pot DNA shuffling method based on type IIs restriction enzymes. PLoS One. 2009;4:1–9. 10.1371/journal.pone.0005553 PMC2677662 19436741

[143] Engler C , Kandzia R, Marillonnet S. A one pot, one step, precision cloning method with high throughput capability. PLoS One. 2008;3:1–7. 10.1371/journal.pone.0003647 PMC2574415 18985154

[144] Engler C , Youles M, Gruetzner R et al. A golden gate modular cloning toolbox for plants. ACS Synth Biol. 2014;3:839–43. 24933124 10.1021/sb4001504

[145] Marillonnet S , Werner S. In: Castilho A, ed. Assembly of Multigene Constructs Using Golden Gate Cloning in Glyco-Engineering: Methods and Protocols. Springer: New York, 2015,269–84. 10.1007/978-1-4939-2760-9_19 26082229

[146] Kuntz M , Chen , Simkin et al. Upregulation of two ripening-related genes from a non-climacteric plant (pepper) in a transgenic climacteric plant (tomato). Plant J. 1998;13:351–61.

[147] Simkin AJ , Qian T, Caillet V et al. Oleosin gene family of *Coffea canephora:* quantitative expression analysis of five oleosin genes in developing and germinating coffee grain. J Plant Physiol. 2006;163:691–708. 16442665 10.1016/j.jplph.2005.11.008

[148] Mukherjee S , Stasolla C, Brule-Babel A et al. Isolation and characterization of rubisco small subunit gene promoter from common wheat (*Triticum aestivum* L.). Plant Signaling and Behavior. 2015;10:1–6. 25713931 10.4161/15592324.2014.989033 PMC4622651

[149] Alotaibi SS , Sparks CA, Parry MAJ et al. Identification of leaf promoters for use in transgenic wheat. Plan Theory. 2018;7:27. 10.3390/plants7020027 PMC6027260 29597282

[150] Alotaibi SS , Alyassi H, Alshehawi A et al. Functional analysis of SBPase gene promoter in transgenic wheat under different growth conditions. Biotechnology. 2019;1:15–23.

[151] Faralli M , Matthews J, Lawson T. Exploiting natural variation and genetic manipulation of stomatal conductance for crop improvement. Curr Opin Plant Biol. 2019;49:1–7. 30851622 10.1016/j.pbi.2019.01.003 PMC6692497

[152] Lawson T , Blatt MR. Stomatal size, speed, and responsiveness impact on photosynthesis and water use efficiency. Plant Physiol. 2014;164:1556–70. 24578506 10.1104/pp.114.237107 PMC3982722

[153] Kapoor L , Simkin AJ, Doss GP et al. Fruit ripening: dynamics and integrated analysis of carotenoids and anthocyanins. BMC Plant Biol. 2022;22:27. 35016620 10.1186/s12870-021-03411-w PMC8750800

[154] Simkin AJ , Kapoor L, Doss CGP et al. The role of photosynthesis related pigments in light harvesting, photoprotection and enhancement of photosynthetic yield in planta. Photosynth Res. 2022;152:23–42. 35064531 10.1007/s11120-021-00892-6

[155] Pazzagli PT , Weiner J, Liu F. Effects of CO_2_ elevation and irrigation regimes on leaf gas exchange, plant water relations, and water use efficiency of two tomato cultivars. Agric Water Manag. 2016;169:26–33.

[156] Reinert RA , Eason G, Barton J. Growth and fruiting of tomato as influenced by elevated carbon dioxide and ozone. New Phytol. 1997;137:411–20. 33863079 10.1046/j.1469-8137.1997.00846.x

[157] Helyes L , Lugasi A, Péli E et al. Effect of elevated CO_2_ on lycopene content of tomato (*Lycopersicon lycopersicum* L. Karsten) fruits. Acta Aliment. 2011;40:80–6.

[158] Hartz TK , Baameur A, Holt DB. Carbon dioxide enrichment of high-value crops under tunnel culture. J Am Soc Hortic Sci. 1991;116:970–3.

[159] Liu J , Peng X, Abdelhakim LOA et al. Carbon dioxide elevation combined with sufficient irrigation and nitrogen fertilization improves fruit quality of tomato grown in glasshouse. Arch Agron Soil Sci. 2021;67:1134–49.

[160] Wei Z , Du T, Li X et al. Interactive effects of elevated CO_2_ and N fertilization on yield and quality of tomato grown under reduced irrigation regimes. Front Plant Sci. 2018;9:1–10. 29636756 10.3389/fpls.2018.00328 PMC5880949

[161] Hicklenton PR , Jolliffe PA. Effects of greenhouse CO_2_ enrichment on the yield and photosynthetic physiology of tomato plants. Can J Plant Sci. 1978;58:801–17.

[162] Fierro A , Gosselin A, Tremblay N. Supplemental carbon dioxide and light improved tomato and pepper seedling growth and yield. Hort Science. 1994;29:152–4.

[163] Yelle S , Beeson RC, Trudel MJ et al. Duration of CO_2_ enrichment influences growth, yield, and gas exchange of two tomato species. J Am Soc Hortic Sci. 1990;115:52–7.

[164] Nilsen S , Hovland K, Dons C et al. Effect of CO_2_ enrichment on photosynthesis, growth and yield of tomato. Sci Hortic. 1983;20:1–14.

[165] Ozcelik N , Akilli M. Effects of CO_2_ enrichment on vegetative growth, yield and quality of greenhouse - grown tomatoes in soil and soilless cultures. Acta Hortic. 1999;491:155–60.

[166] Tripp KE , Peet MM, Pharr DM et al. CO_2_-enhanced yield and foliar deformation among tomato genotypes in elevated CO_2_ environments. Plant Physiol. 1991;96:713–9. 16668247 10.1104/pp.96.3.713 PMC1080835

[167] Li JH , Sagi M, Gale J et al. Response of tomato plants to saline water as affected by carbon dioxide supplementation. I. Growth, yield and fruit quality. J Hortic Sci Biotechnol. 1999;74:232–7.

[168] Porras ME , Medrano E, Lorenzo P et al. Sweet pepper acclimation to variable CO_2_ supply in a Mediterranean greenhouse. Acta Hortic. 2017;1170:797–804.

[169] Milhet Y , Costes C. Effects of CO_2_ nutrition on growth and yield of muskmelon (*Cucumis melo* L.), egg-plant (*Solanum Melongena* L.) and sweet-pepper (*Capsicum annuum* L.). Acta Hortic. 1975;51:201–12.

[170] Garruña-Hernández R et al. Changes in flowering and fruiting of habanero pepper in response to higher temperature and CO_2_. Journal of Food Agriculture and Environment. 2012;10:802–8.

[171] Li X , Kang S, Li F et al. Light supplement and carbon dioxide enrichment affect yield and quality of off-season pepper. Agron J. 2017;109:2107–18.

[172] Imazu T , Yabuki K, Oda Y. Studies on the carbon dioxide environment for plant growth. II. Effect of carbon dioxide concentration on the growth, flowering and fruit setting of eggplant (Solanum melongena L.). Engei Gakkai zasshi. 1967;36:275–80.

[173] Deng X , Woodward FI. The growth and yield responses of fragaria ananassa to elevated CO_2_ and N supply. Ann Bot. 1998;81:67–71.

[174] Mochizuki MJ , Daugovish O, Ahumada MH et al. Carbon dioxide enrichment may increase yield of field-grown red raspberry under high tunnels. Hort Technology. 2010;20:213–9.

[175] Sánchez-Guerrero MC , Lorenzo P, Medrano E et al. Effects of EC-based irrigation scheduling and CO_2_ enrichment on water use efficiency of a greenhouse cucumber crop. Agric Water Manag. 2009;96:429–36.

[176] Sánchez-Guerrero MC , Lorenzo P, Medrano E et al. Effect of variable CO_2_ enrichment on greenhouse production in mild winter climates. Agric For Meteorol. 2005;132:244–52.

[177] Kläring HP , Hauschild C, Heißner A et al. Model-based control of CO_2_ concentration in greenhouses at ambient levels increases cucumber yield. Agric For Meteorol. 2007;143:208–16.

[178] Luomala EM , Kaukoranta T. Altered plant structure and greater yield of cucumber grown at elevated CO_2_ in a semi-closed greenhouse. Acta Hortic. 2008;801:1339–46.

[179] Peet MM . Acclimation to high CO(2) in Monoecious cucumbers : I. vegetative and reproductive growth. Plant Physiol. 1986;80:59–62. 16664607 10.1104/pp.80.1.59 PMC1075056

[180] Raines CA . Improving plant productivity byre-tuning the regeneration of RuBP in the Calvin–Benson–Bassham cycle. New Phytol. 2022;236:350–56. 35860861 10.1111/nph.18394 PMC9833393

